# Does the Habit Theory of Addictions Extend to Disordered Gambling?

**DOI:** 10.1007/s40429-026-00715-5

**Published:** 2026-02-26

**Authors:** Tim van Timmeren, Luke Clark

**Affiliations:** 1 Department of Social, Health, and Organisational Psychology, Utrecht University, Utrecht, The Netherlands; 2 Centre for Gambling Research at UBC, Department of Psychology, University of British Columbia, Vancouver, BC Canada; 3 Djavad Mowafaghian Centre for Brain Health, University of British Columbia, Vancouver, BC Canada

**Keywords:** Gambling, Habits, Goal-directed control, Dual-process model, Behavioral addiction

## Abstract

**Purpose of Review:**

‘Habit theory’ is a pervasive framework that describes addiction as a transition from goal-directed use (e.g. of drugs) to habitual response, accompanied by a neurobiological shift in fronto-striatal brain circuitry. As a theory that has been explored in the context of substance addictions, this article evaluates the conceptual fit of habit theory to gambling behavior and gambling disorder, and summarizes recent empirical evidence.

**Recent Findings:**

Relevant research falls into two main themes. First, studies have compared behavioral markers of habit (e.g. the two-step task, Pavlovian-to-Instrumental Transfer effects) in groups with and without gambling problems. These studies find limited direct support for the hypothesis. Second, psychological research has begun to examine habit-like behaviors in naturalistic gambling. These studies find behavioral expressions consistent with habit formation, primarily during engagement with slot machines, but are yet to test key tenets of habit theory such as insensitivity to outcome devaluation.

**Summary:**

Modern gambling products (e.g. slot machines, in-play sports betting) create rich learning environments that may be highly amenable to habit formation. Further research is needed to develop and validate new tools for testing habit formation and habit strength / persistence in the context of gambling.

## Introduction

Why do some individuals continue gambling despite incurring significant negative consequences that affect their finances, personal relationships, employment, and physical health [1]? Over the past two decades, the ‘habit theory’ of addiction has become a dominant theoretical framework for understanding excessive drug use, drawing on the behavioral science of associative learning, and its underlying neurobiology in terms of fronto-striatal circuitry [2–6]. Habit theory proposes that drug use transitions from an initially goal-directed act, to become habitual with extended drug consumption. The compulsivity of addiction (i.e. its resistance to negative consequences) is thought to arise from this habitual mode of responding. At a neurobiological level, this transition is mediated by a corresponding shift from neural circuitry for goal-directed action to systems that handle habitual responding. This may reflect a shift from prefrontal cortical control to the striatum, and/or *within* the striatum, from the ventral to dorsal striatum [2, 6].

This account was initially developed from behavioral and neural data in animal models (e.g. [7–10]). The past decade has seen an increase in human research on habit, including the operationalizing and quantification of habits, and clinical studies using these tasks in people with substance use disorders. This article considers how habit theory might be further extended to gambling behavior, and to gambling disorder as a case of behavioral addiction. At the same time, we are aware that habit theory has also been subject to robust critique [11–14], and should not be regarded as accepted doctrine for substance addictions. For example, one criticism of habit theory is that drug acquisition often requires careful and motivated planning (e.g. [15]). This caveat also applies when extending the model to gambling; for example, the complexity of traveling to a casino venue, or the resourcefulness in finding new sources of finance for gambling, represent challenges to habit theory.

Although habit-like processes have been acknowledged in some models of behavioral addiction [16–19], we are not aware of any past work that formally considers the central tenets of habit theory in relation to gambling disorder. From one perspective, if drugs interfere with habit processes through their potent pharmacological actions on the dopamine system, then habit theory may have *little* to say about behavioral addictions, in which there is no exogenous chemical agent. On the other hand, modern forms of gambling that are increasingly recognized as most harmful, including slot machines and ‘in-play’ sports betting, can be readily conceptualized in associative learning terms that may be highly amenable to habit formation [20], for reasons we will discuss.

A significant challenge lies in the different usages and definitions of the term ‘habit’. Colloquial references to a ‘drug habit’ or ‘gambling habit’ are widespread, but can obfuscate psychological formulations that aim for greater precision. For instance, in considering the presence of slot machines in public spaces in Finland, Marionneau & Jarvinen-Tassopoulos discuss the “habit-creating nature of gambling” from a sociological perspective that refers to routine action patterns [21]. While this aligns with early social and health psychology definitions of habit based on frequency (e.g. [22]), more recent conceptualizations define automaticity as the critical feature [23]. In contrast, the habit theory of addiction originated in behavioral research on associative learning. We favor this framework as it offers the most precise and rigorous characterization of habits.

The objective of this article is to examine habit theory in relation to gambling, and synthesize current evidence on habits in gambling. We begin by outlining the key features of habit theory as proposed in substance addictions. We highlight a number of relevant conceptual distinctions, including *domain*-*general* tests versus *domain*-*specific* tests of habit, and the related distinction between habit formation as a vulnerability factor for addictions versus habit expression as a mechanism for maintenance or persistence. Next, we describe how habit theory can be applied to gambling and gambling disorder, considering salient differences from drug use, including the role of reward uncertainty in gambling. In presenting extant research on habits in gambling, this work falls into two main themes: clinical studies that compare groups with and without gambling problems on behavioral tests of habit from the associative learning literature, and second, research on habit-like behaviors during engagement in actual gambling, primarily on slot machines. We conclude with future directions and the wider implications of this framework.

## Habit Theory of (Substance) Addictions

According to dual process models of decision making, two systems compete for control over behavior. One of several characteristics that distinguish the two systems is between ‘goal-directed’ and ‘habitual’ behavior [24, 25]. These systems differ in their sensitivity to the expected consequences. Goal-directed behavior is guided by the value of the predicted outcome of each action, i.e. ‘action-outcome’ associations. This system is flexible and sensitive to environmental changes, but it is relatively slow and cognitively demanding. When actions become habitual, these motor patterns become triggered by features of the environment, and detached from the consequences of the actions. In other words, they are driven by ‘stimulus-response’ associations. Once established, habits are rapid, efficient and require minimal cognitive effort, but they are rigid in the face of environmental change. This makes habits efficient for executing routine behaviors, but they can be problematic if the outcomes of those behaviors are no longer desirable.

Like habits, addictive behaviors are often triggered by learned associations to addiction-related ‘cues’ (effectively, conditioned stimuli relevant to the addiction). In formulating what we term the ‘habit theory’ of addiction, Robbins & Everitt [2, 3] proposed that compulsive drug use emerges from a progressive weakening of goal-directed control, and an increasing dependence on stimulus-response habits. To test this specific formulation, researchers must use paradigms that manipulate either the contingency between actions and their outcomes, or the motivational value of the outcome. When an outcome is no longer desirable, goal-directed actions should cease, but habitual actions should persist [24]. This emulates the nature of addiction, in which drug use is difficult to quit and is maintained despite negative consequences.

Habits can also be modulated by addiction-related cues through the phenomenon of Pavlovian-to-Instrumental Transfer (PIT) [2]. In PIT, a classically conditioned stimulus (e.g. a drug cue) invigorates the expression of a learned instrumental response (e.g. drug seeking). With extended drug exposure, this motivational process may further reduce goal-directed learning and enhance habitual learning [2, 26]. The reinforcing properties that conditioned stimuli acquire may also explain how drug seeking is maintained during delays in drug reinforcement [2]. PIT may be an important mechanism in gambling, whereby the energizing effects of conditioned cues in the intervals between gambling episodes may contribute to gambling persistence.

Other work has sought to probe habit theory via its proposed neural substrates, using neuroimaging to compare signals between groups with substance use disorders and healthy controls. At a neural level, the development of addiction is thought to be accompanied by an imbalance in fronto-striatal circuitry, from a system that supports goal-directed behavior to one involved in habitual control [6]. The original formulation characterized this shift from the ventral to the dorsal striatum, supported by evidence from studies of striatal lesions in experimental animals [9], post-mortem histology in people with stimulant addictions [27], and from functional MRI studies of habit learning [28, 29]. Predicated on this work, some studies that detect excessive dorsal striatal activity in groups with addiction infer that this indicates increased habitual processing.

## Key Conceptual Distinctions in Research on Habits

**Fig. 1:**
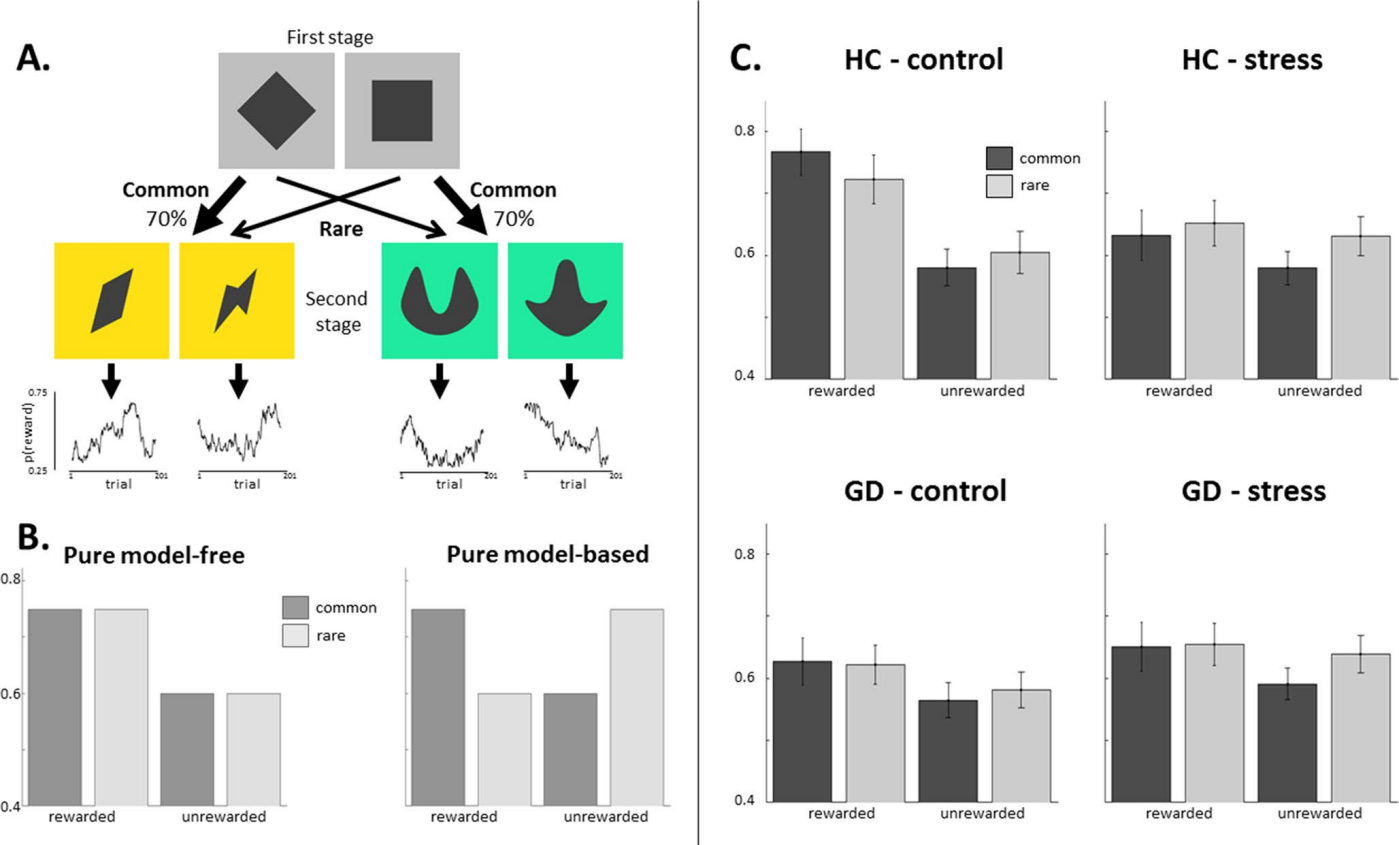
**A**. In the ‘two step’ reinforcement learning task [31], participants first choose between two grey boxes (e.g. the diamond on the left), which would commonly lead them to one second state (e.g. the yellow boxes) and rarely to the other second state (e.g. the green boxes). After selecting one of the second state boxes, they see the outcome of their choice (reward or no reward). Each second state box has a unique reward probability that slowly changes over trials as depicted in the graphs. **B**. The graph on the left depicts a purely model-free learner, whose behavior is solely predicted by whether or not the previous trial was rewarded. A purely model-based learner, on the other hand, takes into account whether a common or rare transition led to the reward. Therefore, receiving a reward after an uncommon transition should increase the propensity to switch and model-based choices are predicted by an interaction between reward and transition. Using this rationale, the logistic regression approach quantifies model-free and model-based control as the main effect of reward and the reward-by-transition interaction, respectively, on stay/switch behavior on each trial. **C**. Across groups and sessions, a main effect of reward and an interaction between reward and transition-type suggested a mixture of both model-free and model-based strategies [46]. Additionally, there was a difference between groups on the main effect of reward and the reward by stress interaction, driven by GD patients showing an overall lower stay probability following rewarding outcomes in the control condition. Figure adapted from van Timmeren, Piray, Goudriaan and van Holst, 2023, ‘Goal-directed and habitual decision making under stress in gambling disorder: An fMRI study’, in *Addictive Behaviors*. Used under a CC-BY-4.0 license

In behavioural research, three tasks are widely used in human studies on habit: the outcome-devaluation procedure (including contingency degradation tasks, as a variant); the so-called ‘two-step task’ (see Fig. 1), which operationalizes habitual versus goal-directed learning in computational terms as model-free versus model-based reinforcement learning; and PIT. These procedures, with the exception of some studies on PIT (e.g. [30]), assess habit as a domain-*general* construct. This is to say, during these tasks, the participant acquires a novel habit, using stimuli and outcomes that are *unrelated* to the addictive behavior. By contrast, a domain-*specific* approach would employ stimuli and/or outcomes that are directly relevant to that addiction. In addictions, cues that are specifically associated with the addictive behavior (i.e. domain-specific) acquire value, and this learning is implicated in the maintenance of addictions [15].

A key question for habit theory is why addicted states would only develop in some individuals, or conversely why many people use substances without developing addictions. Individual differences may exist in the general tendency to form habits. In the context of addictions, these trait-like differences would be expected to shape vulnerability (as opposed to maintenance). These effects should pertain to both substance-related and behavioral addictions. Among healthy people, substantial variability does seem to exist in how easily new activities (e.g. exercise or healthy eating) become habitual [32], and so these effects should be detectable with domain-general tests. Ersche and colleagues developed a Creature of Habit questionnaire to probe these trait-level differences in relation to addiction [33]. Scores were increased among patients with cocaine use disorder, and were correlated with their duration of cocaine use [34]. Likewise, accelerated habit formation offers a possible route to loss of control over gambling.

We note that trait-like differences in habit formation could further interact with the neuroadaptive effects of long-term drug use. Animal studies indicate that addictive drugs may accelerate habit formation, with seeking of both drugs and natural rewards becoming insensitive to reinforcer devaluation [6]. While it is challenging for human (especially clinical) studies to distinguish between pre-existing vulnerability factors and chronic drug effects, clinical studies in substance use disorders do not find consistent support for an imbalance between (domain-general) goal-directed and habitual control [11]. Similarly, for PIT, a recent meta-analysis of 14 studies found that the strength of outcome-specific PIT (see below for further definitions) did not differ significantly between groups with and without substance use disorders [35].

Lastly, impaired top-down processes (dependent on prefrontal cortex) may further reduce inhibitory control over acquired habits [36]. In 2018, a systematic review and meta-analysis examined this possibility in gambling disorder [37], identifying four cognitive domains that may give rise to ‘compulsivity’. While there was consistent evidence for performance deficits on cognitive flexibility, attentional set-shifting, attentional bias in gambling disorder, no empirical studies of habitual control in gambling disorder were identified at that time. Since then, however, considerable progress has been made in the wider operationalizing of habit processes.

## Applying Habit Theory to Gambling Disorder

In considering how habit theory may extend to gambling disorder, there are some important differences to acknowledge between substance use and gambling. Reward uncertainty is a defining feature of gambling, and is likely to be central to the process of reinforcement in gambling. Reward uncertainty, in the form of random ratio schedules and reward anticipation, exerts powerful effects on mesolimbic dopamine signalling (see [38]). However, habitual control develops more rapidly when behavior is reinforced on interval, as opposed to ratio, schedules (e.g. [39]). Habits may benefit from more predictable learning environments; for example, predictable outcomes may facilitate automaticity by reducing attentional focus on the behavior [40]. From this perspective, the unpredictability of gambling outcomes may *hinder*, rather than facilitate habit formation. Although provocative, these issues may be reconciled by noting that the wins are uncertain, but that other reinforcing elements in gambling (e.g. excitement, or escape from distress) are tied to the bets rather than the outcomes, and are therefore largely predictable.

The high level of sensory feedback (e.g. sounds and visuals) during some forms of gambling also makes a powerful substrate for cue learning. While such cues are often win-related, modern (multi-line) slot machines also provide this sensory feedback when the win is less than the bet, i.e. ‘losses disguised as wins’ [41]. This could modulate the ‘experienced’ rate of reinforcement, and, through PIT, may lead to continued gambling despite a negative monetary return. Lastly, recent research shows that goal-directed control is weakened by increasing the interval between an action and the outcome [42]. By extrapolation, habitual processing may be facilitated by delayed outcomes, but in gambling, the most addictive products (e.g., slot machines) tend to be faster games associated with greater immediacy. In summary, there are a number of elements of gambling that may shape habitual processes, including outcome uncertainty, sensory feedback, and outcome delays, but the research characterizing these influences is inconclusive.

## Clinical Studies of Goal-Directed and Habitual Processes

This section considers the empirical evidence for habit theory in relation to gambling disorder. These studies primarily adopt case-control designs, in clinical or community-recruited samples of people with gambling problems. Notably, the three workhorse tasks in research on habits have not been applied uniformly in research on gambling to date. We found no studies using outcome devaluation or contingency degradation tasks in gambling disorder. By contrast, we identified six studies that used the two-step task (see Fig. 1 A) to assess the balance between model-based and model-free learning, and four studies that studied PIT or PIT-like processes.

### Two-Step Task

One study found that participants with gambling problems (*n* = 49) relied less on model-based learning (putatively, less goal directed behaviour) compared to healthy controls (*n* = 33) [43]. This effect was most pronounced following unrewarded outcomes. The group with gambling problems were also faster on these trials, which is potentially an expression of increased impulsivity. Another study, which analyzed behavioral data from a neuroimaging study, did not find significant differences in model-based/model-free learning between two small groups with gambling disorder (*n* = 15) and healthy controls (*n* = 17) [44].

Two studies investigated the effect of acute stress on model-based/model-free learning among people with gambling problems [45, 46]. Stress is an important contextual factor in problem gambling (and other addictions) because it increases craving, and can serve as a trigger for relapse (for review, see [47]). In research in healthy samples, people tend to respond more habitually under stress [48, 49], prompting a hypothesis that problem gambling may be driven by an increased reliance on habits under stress. In the study by Wyckmans and colleagues [45], participants performed the two-step task after a stress induction or control task. The reported analyses focused on whether or not participants showed a cortisol response as a proxy for stress, following previous work (e.g. [48, 50, 51]. Participants were classified as ‘stress responders’ (10 with gambling problems, 15 controls) or ‘non-responders’ (48 gamblers, 43 controls) based on their cortisol change, but independent of the experimental condition (i.e. ‘stress responders’ could be from the non-stress condition, and vice versa). Among non-responders, participants with problem gambling relied less on model-based learning than healthy controls, corroborating earlier results [43]. Amongst stress-responders, there was no difference between groups, and both subgroups relied less on model-based control than non-responding healthy controls. Across participants, a higher cortisol increase was associated with lower model-based learning in controls, but not in the group with gambling problems. These results suggest that the negative effects of stress on model-based learning in healthy participants may be attenuated in people with gambling problems.

A similar question was investigated by van Timmeren et al. [46] using a within-subjects crossover design. People with gambling problems (*n* = 22) and controls (*n* = 20) underwent both acute stress and a control procedure during an fMRI scan. Behavioral analyses indicated the group with gambling problems were more likely to repeat rewarded choices in the control condition, suggestive of increased model-free learning, but not under stress (Fig. 1 C and D). These findings were not supported by the computational modeling approach, however. Bayesian analysis provided evidence against group differences (i.e. supported the null hypothesis), and there were no differences in brain activity between the groups. In summary, these studies provide limited support for the hypothesis that acute stress shifts the balance in favour of habitual control in people with gambling problems.

Finally, two studies assessed the impact of a rather different contextual factor on the two-step task, investigating whether gamblers act more habitually in a gambling environment. In Wagner et al. [52], regular slot machine gamblers (*n* = 30) completed the two-step task on two occasions: in a casino venue that featured a number of active (i.e. in use) slot machines, and in a café (control) environment. In contrast to their hypothesis, the results showed that the gambling context increased a model-based learning strategy and reduced model-free responding, relative to the cafe environment. While striking, it’s unclear how this pattern would compare to healthy non-gamblers. This question was investigated in another study using virtual reality technology to simulate two similar environmental conditions [53]. A group with gambling problems (*n* = 31) and a group of healthy controls (*n* = 29) were again tested in both conditions. Across conditions, the group with gambling problems showed reduced model-based learning compared to controls. Moreover, relative to the cafe context, the casino context made participants in both groups rely more on model-based learning. Although the between-context effects were less pronounced in the virtual reality than the real-world setting, these results broadly extend those by Wagner et al. [52], indicating that the gambling environment or context promotes model-based control in people with a range of gambling involvement.

To summarize, we find three studies that indicate that gamblers show reduced model-based learning (i.e. lower goal-directed responding) relative to controls under baseline conditions [43, 45, 53]. Further research suggests that performance on the two-step task in people with gambling problems does not seem to be differentially affected by acute stress [45, 46] or gambling-related contexts [52, 53]. With this reliance on the two-step task in gambling studies, we note that this task has been subject of recent scrutiny; for example, a recent study indicates that performance is highly sensitive to instructions [54]. This is concerning in case-control design where differences in performance can easily arise from differences in motivation or strategy (broadly, ‘understanding’). Further questions remain about the construct validity of the task, especially in operationalizing habits as model-free responses [55]. Several studies found that insensitivity to outcome devaluation (i.e. habitual responding) correlated with decreased model-*based* control (i.e. reduced goal-directed responding) [56–58]. Thus, while goal-directed learning seems to be affected in gambling disorder [43, 45, 53], the extent to which gambling disorder is marked by increased habit learning is still an open question. This conclusion also applies to the putative shift at the neural level from ventral to dorsal striatum – a central element of habit theory – since the only two neuroimaging studies that we identified [46, 59] did not provide evidence in line with such a shift.

### Pavlovian-to-Instrumental Transfer Tasks

In PIT, a Pavlovian cue (e.g. a stimulus associated with drug use, or gambling) invigorates instrumental responding (e.g. drug seeking, or gambling itself). Current accounts of PIT further distinguish *outcome-specific* transfer, in which cues increase responses for the same outcome (e.g. drug of choice), from *general* transfer, in which the cue increases overall responding (i.e. for both the same and other outcomes). The assessment of PIT in the lab typically involves two training stages of Pavlovian and instrumental learning, followed by a transfer phase to test the influence of reward-related cues on instrumental responding. In research on addictions, most PIT studies have focused on tests of specific transfer, which appears to be largely goal-directed [60]. In a recent meta-analysis, these measures of specific transfer strength did not differ significantly between groups with substance use disorders and healthy controls [35]. If anything, specific transfer was stronger in substance use disorders, against the prediction from habit theory.

Studies in gambling disorder are yet to fully characterize these PIT components within a single task environment, but a number of studies speak to individual components. The presence of audiovisual cues associated with winning have been shown to promote riskier choices in healthy participants [61, 62]. Thus, Pavlovian conditioned cues exert a specific influence on decision-making. Arguably the most clear demonstration of PIT in gambling was conducted by Genauck et al. [63] who studied the effect of gambling-related cues on risky decision making in participants with (*n* = 30) and without (*n* = 30) gambling disorder. Participants could accept or decline coin flip choices offering mixed outcomes (e.g. win 32 euros or lose 11 euros), and the image background displayed either gambling-related, neutral, or emotionally valenced (both positive and negative) cues. Using a machine-learning classifier, the study showed that the individuals with gambling disorder were more likely to accept gambles in the presence of the gambling background cues, compared to both the healthy control group and the other cue conditions. In a follow-up fMRI study, a classification approach was used to extend these findings, showing that a neural PIT signature (consisting of ventral striatum, OFC and amygdala) could also distinguish gamblers from controls [59].

Nevertheless, the gambling cues in these studies may affect decision-making via several cognitive mechanisms, ranging from affective influences via craving to computational processing of value [52, 64]. It remains unclear whether these motivational effects reflect general or specific transfer, although in other behavioral addictions such as gaming, research has shown that specific PIT is enhanced [65, 66]. Together, these results suggest that both discrete and contextual gambling cues can impact decision making, promoting more impulsive and riskier choice, which may have aetiological significance in disordered gambling.

### Habit-Related Processes in Real-World Gambling

**Fig. 2:**
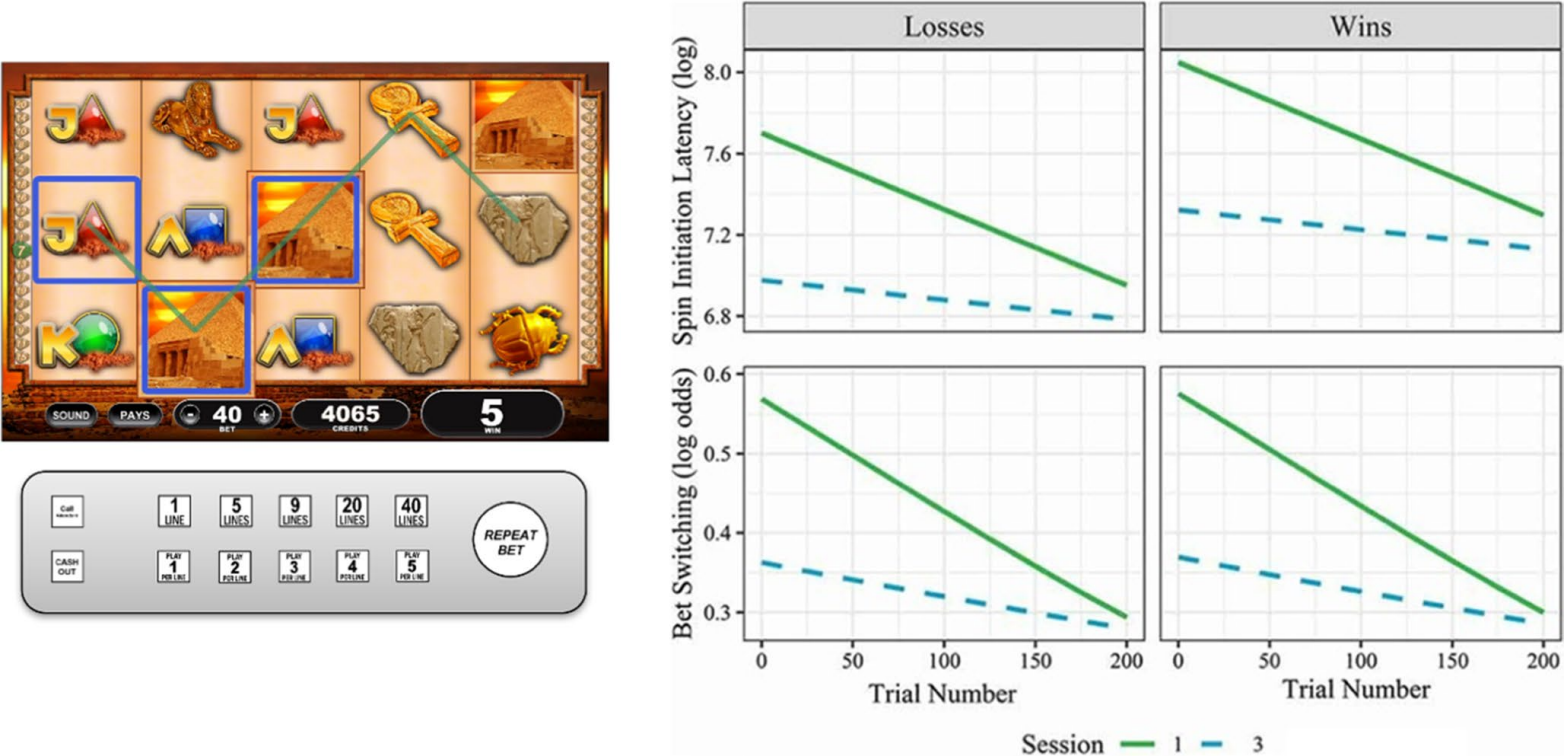
In Ferrari et al [69], participants played a realistic multi-line slot machine game (left panel) that was housed in an authentic slot machine cabinet and used the slot machine button panel as the control interface. Participants played 3 sessions of 200 spins each. In using a simulated game, participants were assigned to pre-configured outcome sequences, so that on each session their financial outcomes fell within a narrow range (that depended on their betting strategy). In the right hand panel, using fixed-effects regression, the predicted probabilities for Spin Initiation Latencies (i.e. the pace of gambling) (upper panel) and the tendency to change one’s bet amount (lower panel) both change with practice, and both within and between sessions. From this figure, it is also clear that initiation latencies are slower after winning outcomes than losing outcomes, i.e. a‘post-reinforcement pause’. (This figure excludes small wins that do not exceed the bet amount, i.e. Losses Disguised as Wins). Figure redrawn with permission from Ferrari, Limbrick-Oldfield & Clark, 2022 ‘Behavioral analysis of habit formation in modern slot machine gambling’, in *International Gambling Studies*, Copyright Taylor-Francis

The studies reviewed so far have used associative learning tasks to probe habits in people with gambling problems. A distinct question is to what extent engagement with a gambling product per se becomes habitual - and if so, whether this behavioral transition is relevant to the development of problem gambling. Forms of gambling often involve distinctive operant properties, such as scratching off the foil barrier on an instant lottery ticket, or ‘shooting’ the dice in craps. For slot machines, as a form of gambling that is well-recognized as among the most harmful forms (e.g. [67, 68], the user interface has changed considerably over the past three decades. The levers on the older ‘one-armed bandits’ have been replaced by a button panel that typically features a large ‘spin’ button (or ‘repeat bet’, in Fig. 2), enabling sessions of gambling to be played via a simple and repetitive action. As slot machines have also become fully computerized (i.e. the spinning reels are now digitally animated), these games have become faster and include high levels of audiovisual features. We posit that these features of modern gambling products offer rich environments for cue associations to form, and could be highly prone to habit formation processes.

Existing theoretical frameworks for problem gambling acknowledge some role for habitual processes. The Pathways Model [70] is best known for proposing three clinical subtypes of people with gambling problems, but within this framework, Pathway 1 describes a psychological sequence that is posited to be common across all people with gambling problems. One of these nodes represents operant and classical conditioning processes, and a further node representing ‘habituation’ is ambiguous in whether it refers to habit formation or the development of tolerance (i.e. that the excitement of gambling diminishes over time, as a potentially non-associative form of learning) (see [69]). If the gradual devaluation of wins via tolerance is an expression of habitual processes, high levels of gambling may persist. Alternatively, if habit formation is accompanied by a reduced sensitivity to losing outcomes, this decoupling of gambling from its aversive consequences could be expressed as loss chasing [43].

Perhaps surprisingly, few studies have examined changes in gambling behaviour as a function of practice or familiarity (see [71, 72]. In an unusual study, Shao et al. [73] studied fMRI neural signals to a simplified slot machine game in participants who were randomized to either practice the task prior to their scan, or complete the task for the first time during their scan. The analyses separated responses to the anticipation (i.e., the reel spin) and the receipt (i.e. outcome) of reward. Compared to the non-practiced group, those with prior task experience showed enhanced anticipatory signaling in brain reward circuitry, and attenuated signaling to winning outcomes. This pattern emulates well-established neurophysiological data for midbrain dopamine neurons as the basis of modern accounts of reinforcement learning [74].

In Ferrari et al. [69], 60 student participants who were deliberately selected as having no prior experience playing slot machines, each attended three sessions spaced roughly one week apart. On each visit, they played 200 spins on a highly realistic simulation of a modern slot machine (see Fig. 2). Two behavioral measures were analyzed to characterize operant changes with practice, both *within* sessions (the 200 spins) and *between* sessions (across the three visits). Motor speed was assessed via spin initiation latencies, which also show a robust ‘post reinforcement pause’ effect following wins. The consistency or rigidity of behaviour was assessed via the likelihood of switching the amount bet. Participants gambled faster, and showed less variability in their betting strategy, as a function of both trial number (within sessions) and visit (between sessions). More unexpectedly, when considering post reinforcement pauses as a function of the size of the win, participants became *slower* with successive sessions of practice. That is to say, participants sped up overall, but simultaneously slowed down after larger wins. While nuanced, this behavioral pattern is quite coherent from a habit perspective, if the lengthening of the post-reinforcement pause is a cue-driven effect (i.e. prompted by win-related audiovisual feedback). This study provides proof-of-concept work for these two behavioural measures as expressions of habit formation in the specific context of slot machine gambling. The next steps would be to validate these variables against contemporary tests of habit formation from associative learning, and investigate group differences as a function of gambling pathology.

### Habit strength, Response Frequency and Automaticity

In other fields like social and health psychology, habits are defined more broadly, focusing on the reported frequency or automaticity of a behavior. Defined in this way, habits can facilitate the development of problematic gambling. On a simple level, it is well known that frequency of gambling is strongly related to problem gambling severity (e.g. [75]). Indeed, future gambling behavior is better predicted by past frequency of gambling, than by satisfaction with gambling [76] or rational explanations for gambling [77]. Thus, for frequent gamblers, behavioral expressions of habit strength are a stronger predictor of harm than intentions. However, gambling frequency is also associated with more *impulsive* gambling [78]. Frequency, as a variable, is likely to be multiply determined, and thus the construct of automaticity may be more informative here [79, 80].

To our knowledge, only one study has used self-reported automaticity as an index of habit strength in relation to gambling behavior [81]. The aim of this study was to investigate whether gambling would be better predicted by people’s intentions, as defined by the Theory of Planned Behavior [82], by past behavior or by self-reported automaticity. Students (*n* = 250) filled out a survey measuring these constructs in addition to attitudes, subjective norms, and perceived behavioral control about gambling). Four weeks later, a subset of the participants (*n* = 180) filled out a survey to assess gambling over the month. Past gambling behavior was associated with higher subjective automaticity, which in turn was negatively related to perceived behavioral control and positively related to future gambling [81]. In other words, subjective automaticity indexing strong habit strength predicted gambling behavior over and above the intention to gamble. This is consistent with a large body of evidence indicating that once behaviors become automatic, they are frequently enacted independently of current goals, making them resistant to change [83].

Lastly, work in the field of information systems has investigated the effectiveness of disruptive features (e.g. pop-up messages) that may act on habits, as interventions for online gambling [84]. Specifically, this study examined the betting histories from over 3,000 gamblers, covering 10 years of data, from a gambling website. About half of these gamblers volitionally activated the disruptive features, while the other half served as a control group. In line with the hypothesis that habits are hard to break, they found that the disruptive features were less effective for more regular gamblers, who also showed stronger repetitive gambling patterns. This pattern was especially evident in sports bettors, who exhibited more repetitive gambling patterns and greater resistance to disruptive features than online casino gamblers. The authors attributed this latter finding to the regular scheduling of sporting events that may encourage frequent site visits and thereby promote habit formation. 

Collectively, studies investigating actual gambling provide preliminary evidence that habits contribute to persistence, but further research is required to further understand whether habit mechanisms contribute to driving and maintaining gambling disorder.

## Future Directions

The habit theory of addiction has become highly influential over the past two decades. In this paper, we have considered how this theory might apply to gambling and gambling disorder. Modern gambling products are designed to invoke fast and repetitive responding with high levels of sensory accompaniment, and in creating these rich environments for operant learning, they may be highly amenable to habit formation. At the same time, habit theory may face theoretical hurdles in its application to gambling, such as evidence that habit formation is greater under conditions of high predictability [40], which clashes with textbook accounts of gambling as intermittent reinforcement. A key question emerging from the existing research is whether clinically-relevant expressions of habit should be domain-general: for example, are individuals who quickly ‘habitize’ many day-to-day behaviors more vulnerable to a range of addictive behaviors that would include gambling? Or, do habit-like processes in (behavioral) addictions follow other forms of addiction-related learning, in being best considered domain-specific [16, 85]? In gambling, for example, associations with gambling-related cues may be the chief driver of excessive consumption. This becomes methodologically important because current paradigms for assessing habits mostly look to build new habits in the lab, using domain-general actions and outcomes. A related question is whether habit *formation* or habit *expression* (i.e. strength and persistence of an existing habit) is more relevant to disordered gambling. Lastly, we note that monetary reinforcers are themselves complex in relation to gambling [86, 87], with ambiguity as to whether money is best considered domain-general or domain-specific.

Turning to the evidence, we identified six studies that used the two-step task to test model-based and model-free reinforcement learning deficits in the context of gambling (disorder). Overall, the findings provide limited support for the narrow hypothesis that gambling disorder is marked by increased model-free (habit) learning, although three studies show reduced model-based (goal-directed) learning [43, 45, 53]. There is some evidence that gambling contexts (e.g. casino environment) may actually increase model-based (goal-directed) responding [52]. Thus, within the confines of the two-step task, current data on gambling disorder speaks against the idea of an increased reliance on habits.

To better understand the role of domain-general habits in gambling, future work would benefit from moving beyond the two-step task. We note the absence of any research using outcome devaluation tests as a particular knowledge gap. This procedure offers a distinct theoretical perspective, although they carry their own methodological issues such as the difficulty in replicating ‘overtraining’ effects [81–83]. We also reviewed a more varied array of studies showing how gambling cues may affect decision-making (both gambling-related and general) [59, 63]. However, it remains unclear whether these effects truly reflect PIT – something that should be addressed in future studies.

Recent studies have begun to examine behavioral components relevant to habit in relation to real-world gambling. Candidate markers based on measures of gambling speed and betting rigidity [69] are both sensitive to repeated practice and become increasingly cue-driven over time. Future studies should examine these markers in combination with the associative learning assays (e.g. outcome devaluation and PIT), to understand the relationship between domain-general and domain-specific processes. The role of habitual tendencies is likely to vary across different forms of gambling; for instance, slot machines are a highly operant and ‘session based’ form of gambling [88] in which habit formation may be a powerful construct. Newer formats such as in-play sports betting may also leverage habit mechanisms via internet and smartphone-based technologies [18], and it will be important to understanding habitual responding across different modes (e.g. online vs. land-based gambling), and different user interfaces (e.g. whether the gambler is using a mouse, keyboard, or touchscreen). As a policy implication, gambling regulators could benefit from enhanced understanding of the habit-forming effects of gambling products and platforms, in order to restrict high-risk features [18, 89]. We suggest that existing research is too limited to identify strong clinical conclusions at the current time, and further work is required before therapeutic techniques like Habit Reversal Training could be utilized. In time, clinical studies may investigate whether the addition of such strategies to standard care improves outcomes, particularly when matched to specific gambler profiles.

## Conclusions

In this article, we have reviewed whether and how the habit theory of substance disorder would apply to gambling disorder, and reviewed the evidence base. Our analysis indicates that there is limited evidence for the idea that gambling disorder is marked by an increased reliance on habits, in part due to a narrow perspective on how habit is operationalized. Notably, most studies have focused on domain-general processes, investigating habit as vulnerability factor. Future research is needed to develop and validate novel tools for testing habit strength and habit acquisition in the context of gambling [90].

## Key References

### Bullet important (•) or very important (••) recent references

•• Ferrari MA, Limbrick-Oldfield EH, Clark L. Behavioral analysis of habit formation in modern slot machine gambling. International Gambling Studies. 2022;00(00):1–20. 

- This study used a highly realistic slot machine simulation to investigate two markers of habit- speed of betting and rigidity in the betting strategy.

• Genauck A, Andrejevic M, Brehm K, Matthis C, Heinz A, Weinreich A, et al. Cue-induced effects on decision-making distinguish subjects with gambling disorder from healthy controls. Addiction Biology. 2019;(July):1–12.

- One of the few studies that directly investigated Pavlovian-Instrumental Transfer in gambling.

•• Lüscher C, Robbins TW, Everitt BJ. The transition to compulsion in addiction. Nature Reviews Neuroscience. 2020 May 30;21(5):247–63. 

- Review paper that provides a recent update of the habit theory of addiction as originally proposed in Everitt & Robbins (2005).

• St Quinton T. Student participation in gambling: the role of social cognition, past behaviour, and habit. Psychology, Health & Medicine 2022;27:1774–81. 

- This study investigates the role of automaticity in gambling via the Theory of Planned Behavior. Results indicate that automaticity predicts future gambling behavior over and above the intention to gamble, suggesting a role for habit in the persistence of gambling.

• van Timmeren T, Piray P, Goudriaan AE, van Holst RJ. Goal-directed and habitual decision making under stress in gambling disorder: An fMRI study. Addictive Behaviors. 2023 May 1;140:107628.

-  This FMRI study tested the effect of acute stress on model-based/ model-free learning following stress-induction and a control condition.

• Wagner B, Mathar D, Peters J. Gambling Environment Exposure Increases Temporal Discounting but Improves Model-Based Control in Regular Slot-Machine Gamblers. Computational Psychiatry. 2022 Jul 5;6(1):142–65. 

- In this preregistered study, regular gamblers performed the two-step task in a casino and a neutral environment (cafe). In contrast to the hypothesis, model-based learning was improved in the gambling context.

• Wyckmans F, Otto AR, Sebold M, Daw N, Bechara A, Saeremans M, et al. Reduced model-based decision-making in gambling disorder. Scientific Reports. 2019;9(1):1–10. 

- This two-step study in gambling disorder found decreased model-based learning compared to healthy controls. Interestingly, this effect was most pronounced following unrewarded outcomes.

## Data Availability

No datasets were generated or analysed during the current study.
